# Giant breast tumors: Surgical management of phyllodes tumors, potential for reconstructive surgery and a review of literature

**DOI:** 10.1186/1477-7819-6-117

**Published:** 2008-11-11

**Authors:** Margaret I Liang, Bhuvaneswari Ramaswamy, Cynthia C Patterson, Michael T McKelvey, Gayle Gordillo, Gerard J Nuovo, William E Carson

**Affiliations:** 1 The Ohio State College of Medicine, Columbus, Ohio, USA; 2 The Ohio State University Department of Haematology-Oncology, Arthur G. James Cancer Hospital and Richard J. Solove Research Institute, Division of Internal Medicine, Columbus, Ohio, USA; 3 The Ohio State University Division of Dermatology, Columbus, Ohio, USA; 4 The Ohio State University Division of Plastic Surgery, Columbus, Ohio, USA; 5 The Ohio State University Department of Pathology, Columbus, Ohio, USA; 6 The Ohio State University Department of Surgery, Arthur G. James Cancer Hospital and Richard J. Solove Research Institute, Division of Surgical Oncology, Columbus, Ohio, USA

## Abstract

**Background:**

Phyllodes tumors are biphasic fibroepithelial neoplasms of the breast. While the surgical management of these relatively uncommon tumors has been addressed in the literature, few reports have commented on the surgical approach to tumors greater than ten centimeters in diameter – the giant phyllodes tumor.

**Case presentation:**

We report two cases of giant breast tumors and discuss the techniques utilized for pre-operative diagnosis, tumor removal, and breast reconstruction. A review of the literature on the surgical management of phyllodes tumors was performed.

**Conclusion:**

Management of the giant phyllodes tumor presents the surgeon with unique challenges. The majority of these tumors can be managed by simple mastectomy. Axillary lymph node metastasis is rare, and dissection should be limited to patients with pathologic evidence of tumor in the lymph nodes.

## Background

The phyllodes tumor, originally described by Johannes Muller in 1838, has presented a diagnostic and treatment dilemma for physicians since its original description. Classically, the name cystosarcoma phyllodes was assigned because of the tumor's fleshy appearance and tendency to contain macroscopic cysts. The term, however, is a misnomer as these tumors are usually benign. Phyllodes tumor is the currently accepted nomenclature according to the World Health Organization (WHO).

While the surgical management of the phyllodes tumor has been addressed many times in the literature, few reports have specifically commented on the giant phyllodes tumor, an entity that presents the surgeon with several unique management problems. The median size of phyllodes tumors is around 4 cm [1]. Twenty percent of tumors grow larger than 10 cm, the arbitrary cut off point for the designation as a giant tumor. These tumors can reach sizes up to 40 cm in diameter [2]. We will review two recent cases at The Ohio State University that had pre-operative diagnoses of giant phyllodes tumor and discuss the surgical techniques employed, including the reconstructive options.

## Methods

We performed a retrospective chart review of patients treated for giant phyllodes tumors at The Ohio State University Medical Center between the years 1999–2001. A Medline search for articles in the English language using the key words phyllodes tumor and cystosarcoma phyllodes was also conducted.

## Case presentation

### Case 1

Patient A is a 64 year old white female who presented with a large right breast mass. The mass had been present for at least 2 years, and the breast was swollen, streaked with grey and blue, and mildly tender. There was no personal or family history of breast cancer. Her past medical history was significant for a hysterectomy and oophorectomy at 46 years of age. Her first menstrual period was at age 13, and she had never been pregnant.

**Figure 1:**
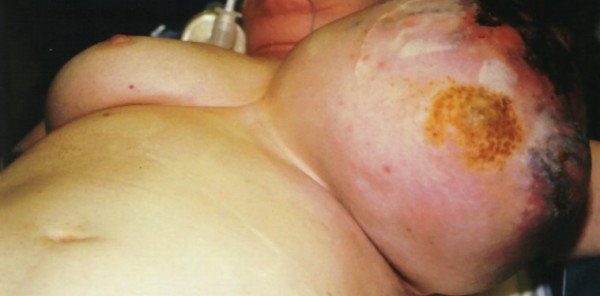
Case 1: The mass measured 36 × 30 cm with the characteristic bluish discoloration of the skin with nipple excoriation.

Physical exam revealed a well-nourished female with an obvious mass of the right breast. The mass measured 36 × 30 cm at the time of presentation. The skin of the breast was blue at the apex of the mass, and the nipple was massively enlarged and excoriated (Fig 1). On initial presentation, there was no evidence of skin breakdown, but by the time of surgery, the patient had experienced loss of skin integrity. The contralateral breast was of normal size, with no significant masses on palpation. There was no palpable adenopathy in either of the axillary basins.

Fine needle aspiration was performed during the initial visit and revealed highly atypical cells suspicious of a malignant neoplasm. Core tissue biopsy showed mixed epithelial-stromal proliferation suggestive of a phyllodes tumor. CT scans of the chest, abdomen, and head showed no evidence of distant metastasis but did suggest invasion of the tumor into the chest wall. For this reason, initial surgical management only involved tumor resection, and breast reconstruction was deferred.

**Figure 2:**
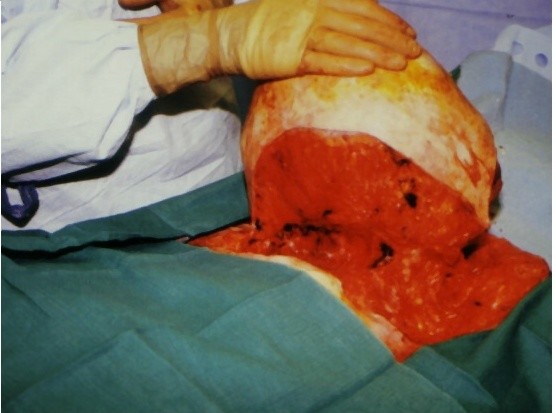
Case 1: Intra-operative photo revealing dissection of the tumor with no invasion of the deeper chest wall.

**Figure 3:**
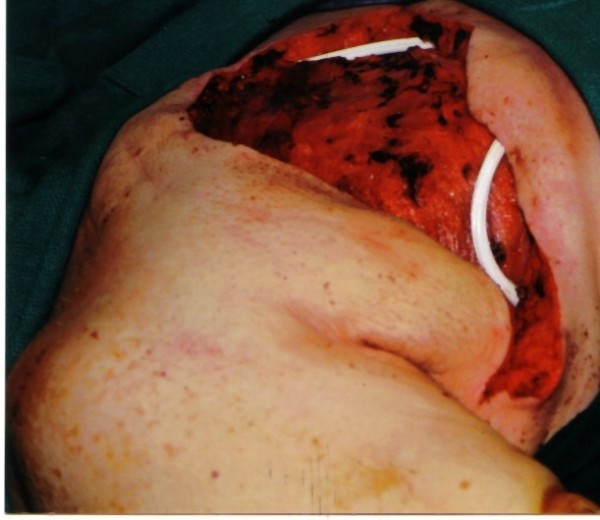
Case 1: Intra-operative photo after tumor resection with placement of two #19 Blake drains under the superior and inferior flaps.

Right simple mastectomy was performed. Superior and inferior skin flaps were designed to allow skin approximation and closure after removal of the large tumor. These flaps included skin directly overlying the tumor that appeared normal (Fig 2). The superior flap was raised to the level of the clavicle. Dissection revealed that the blood supply to the tumor was derived largely from collateral vessels arising from the skin. These vessels were large and friable, yet easily managed using standard techniques. The inferior flap was then raised, demonstrating tumor that was partly adherent to the inferior aspect of the pectoralis major muscle. No blood supply originated from the muscle. A portion of the pectoralis major muscle was excised with the tumor, and no invasion of deeper chest wall structures was noted. No lymphadenopathy was appreciated; therefore, an axillary lymph node dissection was not pursued. Two #19 Blake drains were placed beneath the superior and inferior flaps, followed by approximation of the flaps and skin closure (Fig 3).

**Figure 4:**
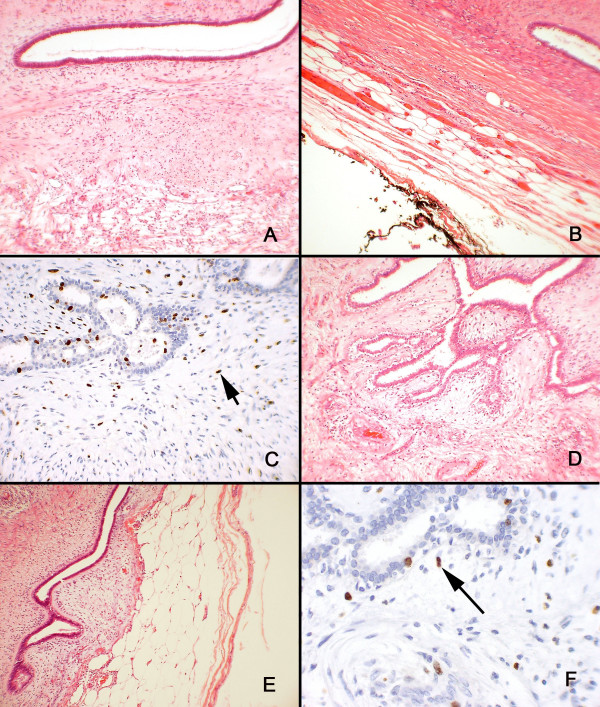
Case 1: A) Large, simple ducts were surrounded by a uniform, bland stroma in this tumor, which measured 30.0 × 25.0 × 20.0 cm *ex vivo*. B) The tumor had negative margins of resection that ranged from 0.3 to 1.0 cm. C) The Ki67 proliferation index for the tumor from patient A was 5 for the epithelial component and 13 for the stromal component. Case 2: D) Large, branching ducts were surrounded by a uniform, bland stroma; areas of hyalinization and myxoid change were rare in this 10.0 x 8.0 x 5.0 cm tumor. E) The tumor had negative margins of resection that ranged from 0.3 to 1.0 cm. F) The Ki56 proliferation index for the tumor from patient B was 0.8 for the epithelial component and 4 for the stromal component.

The pathologic findings of this procedure were consistent with benign phyllodes tumor. The tumor measured 30.0 × 25.0 × 20.0 cm *ex vivo*. Microscopic sections demonstrated large, simple ducts surrounded by a uniform, bland stroma (Fig 4D). The margin of resection was negative for the tumor with a tumor-free zone that ranged from 0.3 to 1.0 cm (Fig 4E). The Ki67 proliferation index of the tumor from patient A was 5 and 13 for the epithelial and stromal component, respectively (Fig 4F). The epithelial component Ki67 index from this specimen is typical of adenomas, whereas the index of 13 for the stromal component is consistent with benign yet brisk stromal proliferation. Mitoses were less than 3 per 10 high power field. Three axillary lymph nodes were present within the mastectomy specimen, and none of these showed evidence of malignancy. The patient is currently over 7 years post-surgery and has shown no evidence of local recurrence or distant metastasis.

### Case 2

Patient B is a 70 year old white female who presented with a large left breast mass. The patient was unsure how long the lesion had been present. Nonspecific findings consistent with a fibroadenoma were noted in the same region on a mammogram obtained 5 years previous to her presentation. Her past medical history was significant for hysterectomy at age 52. She had no personal history of cancer, although her family history was significant for lung and pancreatic cancers. She had used an estrogen and progesterone combination for hormone replacement in the past, the duration of which was unclear.

On physical examination, the patient's left breast had a multilobulated and relatively firm mass that measured approximately 12 cm in diameter and essentially replaced the entire breast. No cervical, supraclavicular, or axillary lymphadenopathy was noted. The contralateral breast showed no signs of a mass. Core tissue biopsy taken at the time of presentation suggested a diagnosis of cellular fibroadenoma or phyllodes tumor. Pre-operative CT scan revealed a 7.5 × 11 cm mass in the anterior portion of the breast, with no apparent mediastinal, lung, neck, or axillary lymphadenopathy.

Left modified radical mastectomy with dissection of level I and level II lymph nodes was performed. A right mastopexy was performed for purposes of symmetry. An elliptical incision encompassing the entire mass and the overlying skin was made. Collaterals in the skin supplied the tumor, and no deep invasion was identified. The superior and inferior skin flaps included skin that had been overlying the tumor. The tumor was excised along with the pectoralis muscle fascia. Axillary dissection was undertaken because of the presence of palpable level II nodes intra-operatively. A tissue expander was placed before final closure, as the patient desired reconstruction.

The pathologic findings of this procedure were consistent with a benign phyllodes tumor. The tumor measured 10.0 × 8.0 × 5.0 cm *ex vivo*. Microscopic sections showed large branching ducts surrounded by a uniform, bland stroma (Fig 4D); areas of hyalinization and myxoid change were rare. As with patient A, the margin of resection was negative for the tumor with a tumor-free zone that ranged from over 0.3 to 1.0 cm (Fig 4E). The Ki67 proliferation index of the tumor from patient B was 0.8 and 4 for the epithelial and stromal component, respectively (Fig 4F). No significant cytologic atypia or mitotic activity was noted. Sixteen lymph nodes were obtained, all of which were benign.

The patient had an unremarkable post-operative course and was able to start tissue expansion 19 days after her surgery. She had exchange of her tissue expander for a permanent implant 6 months after her mastectomy. She had an uneventful recovery from these surgical procedures and is currently 6 years post-surgery without complication.

## Discussion

Phyllodes tumors are fibroepithelial neoplasms with epithelial and cellular stromal components, the latter of which represents the neoplastic process [3]. The presence of an epithelial component differentiates the phyllodes tumor from other stromal sarcomas. They make up 0.3 to 0.5% of female breast tumors [1] and have an incidence of about 2.1 per million, the peak of which occurs in women aged 45 to 49 years [4,5]. The tumor is rarely found in adolescents and the elderly [6,7].

Classically, patients present with a firm, mobile, well-defined, round, macrolobulated, and painless mass. There are no pathognomonic mammographic or ultrasound features. Hence the phyllodes tumor can be extremely difficult to differentiate from a fibroadenoma, which is sometimes treated with a non-operative approach [3]. For this reason, early diagnosis of the phyllodes tumor is crucial so that the correct management of the tumor, which often does include surgery, can be pursued as early as possible. This may also prevent the growth of phyllodes tumors into giant ones, such as in the two cases described in our report. In a series of 106 patients by Chua *et al.*, 71% of patients with a post-operative diagnosis of phyllodes tumor had a presumptive diagnosis of fibroadenoma at the time of surgery [8]. Another series showed a pre-operative diagnosis of phyllodes tumors in only 10 to 20% of patients [1].

A variety of techniques have been utilized to improve the pre-operative diagnosis of phyllodes tumors. Cole-Beuglet *et al. *performed a retrospective study on 8 cases of histologically proven phyllodes tumors that were evaluated by mammography and ultrasound. They determined that while certain ultrasound findings (low-level internal echoes, smooth walls, good through transmission, and smooth margined fluid-filled clefts in a predominantly solid mass) may suggest a phyllodes tumor, there is no consistent and reliable way to distinguish between phyllodes tumors and other benign appearing tumors on ultrasound or mammography [9]. In a recent review examining the use of ultrasound in the diagnosis of phyllodes tumors, Chao *et al. *identified three sonographic features that are characteristic of these tumors: well-circumscribed, lobulated masses, heterogeneous internal echo patterns, and a lack of microcalcifications [10]. In addition, the authors shed light on the pre-operative distinction between fibroadenomas and phyllodes tumors. Patients with fibroadenomas are generally younger than the patients with phyllodes tumors; fibroadenomas have a larger ratio of length to anteroposterior diameter; and phyllodes tumors are generally larger than fibroadenomas [10]. Another group investigated the possibility of establishing a pre-operative diagnosis of malignant or benign phyllodes tumor through the use of color Doppler ultrasound. They concluded that although several ultrasonographic features are characteristic of a malignant phyllodes tumor, a histologic specimen should be obtained for definitive diagnosis. The features that suggested a malignant behavior were "marked hypoechogenicity, posterior acoustic shadowing, and higher values of RI (resistance index), PI (pulsatility index), and Vmax (systolic peak flow velocity) [11].

Another potentially useful diagnostic modality is magnetic resonance imaging (MRI). One article discussed the use of MRI in characterizing benign phyllodes tumors. Findings consistent with a benign phyllodes tumor included a lobulated or polygonal shape with smooth borders, cystic or septated features, and a gradual or rapid pattern of time-signal intensity curve [12]. In a recent correspondence, Cheung *et al. *discussed the pathological features typical of phyllodes tumors and how they are manifested in MRI. The authors went so far as to suggest that the findings of "characteristic leafy internal morphology, best shown on subtraction MRI, which highlighted the enhancing cotyledonous solid portions within irregular blood-filled cystic spaces" are pathognomonic for a phyllodes tumor [13].

Fine needle aspiration (FNA) has also been proposed as a method to improve pre-operative diagnosis; however, existing reports are not promising. Salvadori *et al. *found the FNA to be diagnostic in only 4 of 30 cases [5]. Other investigators have obtained similar results and have concluded that FNA is usually non-diagnostic [14]. The difficulty in diagnosing the phyllodes tumor by FNA is compounded by the fact that it shares many cytologic features with fibroadenoma [15,16].

Core tissue biopsy is an attractive alternative to FNA, and several authors have suggested its use as a diagnostic procedure [8,17]. Interestingly, patient A had an initial non-diagnostic FNA followed by a core tissue biopsy suggestive of phyllodes tumor. Patient B had a core tissue biopsy only, which provided a preliminary diagnosis of phyllodes tumor. We believe that core tissue biopsy represents the preferred means of pre-operative diagnosis for giant breast tumors, and the histologic information gained from this procedure is important in guiding surgical treatment.

Phyllodes tumors are divided into benign, borderline, and malignant histotypes based on the microscopic appearance of the stromal component. Approximately 15 to 30% of all phyllodes tumors are classified as malignant [5,18-20]. Histologic appearance may not, however, correlate with clinical behavior [17,18,20,21], as both malignant and borderline tumors have been shown to be capable of metastasizing. Reinfuss *et al.*, using histotype criteria developed by Azzopardi and Salvalori *et al. *[5], showed that the histotype of the tumor was an independent prognostic factor, with 5-year survivals of 95.7% for benign tumors, 73.7% for borderline tumors, and 66.1% for malignant tumors [2]. A study by Chaney *et al.*, which combined the benign and borderline tumors into a single category, found 5-year survival rates of 91% for benign tumors and 82% for malignant tumors. Ten-year survival rates, however, dropped to 79% and 42%, respectively [20]. A recent review and clinical follow-up of 33 cases concluded that histopathological classification is the strongest prognostic factor for this disease [22]. Others have failed to duplicate the correlation between histotype and survival [17]. Metastasis is seen in 25 to 31% of malignant tumors [1,20], but only in 4% of all phyllodes tumors [5]. The most common sites for metastasis include the lungs, bone, liver, and distant lymph nodes. Skin involvement with tumor does not appear to be a predictor of metastasis [23]. While the use of chemotherapy and radiotherapy have shown promise in a few small trials, their role in the treatment of metastatic phyllodes tumors remains unproven. Hormonal therapy has also been attempted, but with limited efficacy [20,24-26]. Pathologic factors associated with poor prognosis include greater than 3 mitotic figures per high power field, infiltrating margins, severe atypia, stromal overgrowth, stromal component other than fibromyxoid, and tumor necrosis [20,27-30]. Hawkins *et al. *reported a strong correlation between stromal overgrowth and metastasis, finding that 72% of tumors with stromal overgrowth will eventually develop a metastasis [27]. Variables such as age, symptom duration, and tumor growth rate are not of prognostic value [27-30]. Phyllodes tumors do not typically metastasize via the lymphatics. About 20% of patients have palpable axillary lymph nodes on presentation, but only 5% of these show histologic evidence of metastasis upon pathologic examination. Of the phyllodes tumors with a malignant histotype, up to 15% will metastasize to the axilla [30]. In a 1972 retrospective report, Kessinger *et al. *found that all metastatic lesions described in the literature contained only stromal elements. No malignant epithelial elements were observed. Since most sarcomas metastasize hematogenously, this finding may explain why axillary metastasis is so rare [24]. Palpable lymphadenopathy is typically attributed to the patient's immune response to tumor necrosis. The rare patient who does have lymph node metastases tends to have a poor prognosis [30]. Observing the rarity of lymph node involvement, most authors have concluded that removal of axillary lymph nodes is not warranted unless there are palpable nodes [2,14,20,29,31,32]. Data regarding sentinel lymph node biopsy in phyllodes tumors are lacking. In patient A, 3 lymph nodes were included as part of the mastectomy specimen. In patient B, palpable nodes were present, therefore axillary dissection was performed. However, neither patient showed evidence of tumor spread to the lymph nodes. Theoretically, the axillary nodal basin can be evaluated with sentinel lymph node biopsy and subsequent frozen section in patients that have clinically negative axillary nodes. However, patients with giant phyllodes may have clinically enlarged axillary lymph nodes that may be suspicious for metastatic disease. Sentinel lymph node biopsy may not be accurate in these patients and the surgeon may be forced to proceed with axillary lymph node dissection.

About 20% of phyllodes tumors would be considered giant, or greater than 10 cm in maximum diameter [2]. As mentioned before, the importance of this cut-off value has been disputed. There is a continuing debate that exists over the prognostic significance of tumor size [23,26,28,32]. Thus, appropriate cut off values for tumor size and associated prognosis have never been defined [18]. There is also disagreement as to whether malignant histology correlates with size. Some investigators show that malignant tumors tend to be larger than benign ones [8,26], while others have failed to duplicate this association [2,20].

Surgical management of the phyllodes tumor has also been a source of debate over the years. Some authors have argued for simple mastectomy for phyllodes tumors because of the risk of local recurrence after more conservative procedures [23,24,30,31]. However, studies have shown no differences between breast conserving surgery versus mastectomy in terms of metastasis-free survival or overall survival, despite the higher incidence of local recurrence that comes with breast conserving surgery [28]. Most experts currently advocate that surgeons obtain at least 1 cm margins on primary excision or re-excision of a tumor removed with close margins, as long as the tumor to breast size will permit [1,2,5,8,14,17,20,30,33]. However, an excision with the required margins is often impossible in giant phyllodes tumors such as the cases reported here. Mastectomy should be reserved for larger tumors [18,26] and should be considered in recurrent tumors, especially of the malignant histotype [5,33]. Specifically, in cases in which phyllodes tumors have gone undetected and developed into giant phyllodes tumors, particular emphasis should be placed upon complete removal of all visible tumor. Local recurrence in phyllodes tumors has been associated with inadequate local excision and various histological characteristics, including mitotic activity, tumor margin, and stromal cellular atypia [34]. Because of the danger of recurrence that accompanies an incomplete resection or a resection characterized by close margins, the surgeon is often faced with the need for mastectomy for phyllodes tumors that are greater than 10 cm. Depending on the size of the breast and the location of the phyllodes tumor, mastectomy may also be required for tumors that are between 5 and 10 cm in diameter [35]. It should be emphasized that by the time a phyllodes tumor becomes giant, there is no guarantee that the remaining breast tissue has not been infiltrated by tumor cells. Hence, the emphasis should be on complete extirpation of all visible tumor and breast tissue during mastectomy. If all breast tissue has been removed, and all tumor infiltrated soft tissues have been removed, then the tumor is unlikely to recur locally.

In both of our cases, mastectomies were considered the appropriate surgical procedure because of the large size of the tumors when compared to the overall amount of breast tissue. This procedure provides the best opportunity for obtaining clear margins, thereby reducing the likelihood of tumor recurrence. We found that normal skin overlying the tumor could be preserved with the expectation of close but negative margins. This approach permitted skin closure without the need for split thickness skin grafting in patient A and allowed placement of a tissue expander in patient B. The ability to preserve the overlying skin flaps is an important consideration in the surgical management of giant phyllodes tumor as it generally allows for a more satisfying cosmetic result.

Chest wall invasion appears to be an uncommon event with phyllodes tumors. Reinfuss *et al. *reported that 2.4% of phyllodes tumors in their series had clinically recorded infiltration into the pectoralis major muscle [2]. Moore and Kinne recommend extended excision of involved pectoralis muscle, followed by reconstruction of the chest wall with Marlex mesh and methylmethacrylate [32]. Some have recommended the consideration of post-operative radiation for cases of chest wall infiltration [14]. Although patient A showed evidence of pectoralis major muscle invasion, the involvement was not extensive and was easily managed by excision of a portion of the muscle. No invasion of deeper structures was noted in the other case.

Foreknowledge of the location of the tumor's blood supply can be vital information when removing large tumors. Little has been written on the subject with regard to giant phyllodes tumors or breast cancers in general. A case report by Jonsson and Libshitz documented the angiographic pattern of a 25 cm phyllodes tumor. The tumor was hypervascular with irregular and tortuous arteries. Blood supply to the tumor was via one large and several smaller perforating anterior branches of the internal mammary, lateral thoracic, acromio-thoracic arteries, and branches of the axillary artery [36]. We found that the giant tumors in the present report derived the majority of their blood supply from skin collaterals. Thus, the surgeon can expect the majority of blood loss during resection to come from the creation of the skin flaps. In this situation, the surgeon need not routinely obtain an angiogram.

In general, immediate breast reconstruction can be performed at the time of mastectomy for phyllodes tumors [14]. Mandel *et al. *reported a case in which subcutaneous mastectomy was performed for a large phyllodes tumor, followed by immediate implantation of a breast prosthesis. They cite minimal interference with the detection of recurrent lesions and the minimization of emotional distress as advantages to the procedure [37]. Orenstein and Tsur described a similar case in an adolescent female in which a silicon implant was placed under the pectoralis major, where it would not impair the recognition of recurrent disease [38].

Local recurrence rates for phyllodes tumors are 15 to 20% and are correlated with positive excision margins, rather than with tumor grade or size [1,8,14,31]. Other studies have shown a higher risk of local recurrence in borderline and malignant tumors. In a series of 21 patients by Salvadori *et al*., 51 patients were treated with breast conserving surgery (enucleations, wide excisions), and 14 of the tumors recurred locally. In contrast, the 20 patients treated with mastectomy (subcutaneous, modified radical, or radical) showed no evidence of local recurrence [5]. Importantly, there is no contraindication to immediate reconstruction after mastectomy in cases of giant phyllodes tumor, and this decision can be made solely based upon patient peference [37,38].

## Conclusion

In summary, management of the giant phyllodes tumor presents the surgeon with unique challenges. Diagnostically, we believe that core tissue biopsy represents an attractive means for pre-operative diagnosis and aids in the differentiation of phyllodes tumors from fibroadenomas. The majority of these tumors can be managed by simple mastectomy. Axillary lymph node metastasis is rare, and dissection should be limited to patients with pathologic evidence of tumor in the lymph nodes. There is no contraindication to immediate reconstruction after mastectomy.

## Competing interests

The authors declare that they have no competing interests.

## Authors' contributions

CCP, GG and MIL helped with writing and editing. BR and MTM helped with researching, writing, and editing. GN performed, reviewed, and interpreted all the pathology slides and reports. GG and WC were surgeons involved in the cases. WC developed and oversaw the project.

## Consent

Written informed consent was obtained from the patients for publication of these case reports.
